# Clinical predictors of extubation failure in postoperative critically ill patients: a post-hoc analysis of a multicenter prospective observational study

**DOI:** 10.1186/s12871-025-02996-1

**Published:** 2025-03-15

**Authors:** Jun Hattori, Aiko Tanaka, Junko Kosaka, Osamu Hirao, Nana Furushima, Yuichi Maki, Daijiro Kabata, Akinori Uchiyama, Moritoki Egi, Hiroshi Morimatsu, Satoshi Mizobuchi, Yoshifumi Kotake, Ayumi Shintani, Yukiko Koyama, Takeshi Yoshida, Yuji Fujino

**Affiliations:** 1 Faculty of Medicine, Osaka University, 2-15 Yamadaoka, Suita, 565-0871 Osaka Japan; 2 Department of Anesthesiology and Intensive Care Medicine, Osaka University Graduate School of Medicine, 2-15 Yamadaoka, Suita, 565-0871 Osaka Japan; 3 Department of Intensive Care, University of Fukui Hospital, 23-3 Matsuoka Shimoaizuki, Eiheiji-cho, Yoshida, 910-1193 Fukui Japan; 4 Department of Anesthesiology and Resuscitology, Okayama University Hospital, 2-5-1 Shikata-cho, Kita- ku, Okayama, 700-8558 Japan; 5 Department of Anesthesiology, Osaka General Medical Center, 3-1-56 Bandai-Higashi, Sumiyoshi-ku, Osaka, 558-8558 Japan; 6 Department of Anesthesiology and Intensive Care Medicine, Kobe University Hospital, 7-5-2 Kusunoki- cho, Chuo-ku, Kobe City, 650-0017 Japan; 7 Department of Anesthesiology, Toho University Ohashi Medical Center, 2-22-36, Ohashi, 153-8515 Meguro, Tokyo Japan; 8 Center for Mathematical and Data Science, Kobe University, 1-1 Rokkodai-cho, Nada-ku, Kobe, 657-8501 Hyogo Japan; 9 Department of Anesthesia, Kyoto University Hospital, 54 Shogoin-kawahara-cho, Sakyo-Ku, Kyoto, 606- 8507 Japan; 10 Department of Medical Statistics, Graduate School of Medicine, Osaka Metropolitan University, 1-4-3, Asahi-machi, Abeno-ku, Osaka, 545-8585 Japan

**Keywords:** Reintubation, Extubation failure, Endotracheal suctioning, Postoperative patient, Clinical predictor, Critical care

## Abstract

**Background:**

Postoperative patients constitute majority of critically ill patients, although factors predicting extubation failure in this group of patients remain unidentified. Aiming to propose clinical predictors of reintubation in postoperative patients, we conducted a post-hoc analysis of a multicenter prospective observational study.

**Methods:**

This study included postoperative critically ill patients who underwent mechanical ventilation for > 24 h and were extubated after a successful 30-min spontaneous breathing trial. The primary outcome was reintubation within 48 h after extubation, and clinical predictors for reintubation were investigated using logistic regression analyses.

**Results:**

Among the 355 included patients, 10.7% required reintubation. Multivariable logistic regression identified that the number of endotracheal suctioning episodes during the 24 h before extubation and underlying respiratory disease or pneumonia occurrence were significantly associated with reintubation (adjusted odds ratio [OR] 1.11, 95% confidence interval [CI] 1.05–1.18, *p* < 0.001; adjusted OR 2.58, 95%CI 1.30–5.13, *p* = 0.007). The probability of reintubation was increased significantly with the higher frequency of endotracheal suctioning, as indicated by restricted cubic splines. Subgroup analysis showed that these predictors were consistently associated with reintubation regardless of the use of noninvasive respiratory support after extubation.

**Conclusions:**

Endotracheal suctioning frequency and respiratory complications were identified as independent predictors of reintubation. These readily obtainable predictors may aid in decision-making regarding the extubation of postoperative patients.

**Supplementary information:**

The online version contains supplementary material available at 10.1186/s12871-025-02996-1.

## Background

Extubation failure, reintubation, is a crucial aspect affecting patient prognosis in intensive care [1]. International guidelines recommend spontaneous breathing trial (SBT) as a standard evaluation prior to extubation [2, 3]. However, extubation failure still occurs in approximately 10% of patients who are extubated after successful SBT [4, 5]. Extubation failure has been associated with increased incidence of ventilator-associated pneumonia, prolonged duration of mechanical ventilation, and longer intensive care unit (ICU) and hospital stays, contributing to increased mortality [1, 6, 7, 8]. Given that extubation failure deteriorates patient prognosis, predictors of extubation outcomes have been investigated and risk factors including age, Glasgow Coma Scale (GCS) score, and PaO_2_:FiO_2_ have been indicated [9, 10, 11]. Furthermore, prediction models incorporating multiple clinical predictors and imaging diagnostics, such as diaphragmatic ultrasonography, have been proposed [12, 13, 14, 15, 16, 17, 18]. However, the guidelines for extubation in intensive care do not specify predictors of extubation [2, 19], and definitive methods for predicting reintubation, particularly in postoperative patients, have yet to be established [13, 20].

Postoperative patients constitute a significant portion of intensive care patients, accounting for 21 to 60% of all critically ill patients [21, 22, 23]. General anesthesia reduces muscle tone, resulting in a decrease in thoracic and airway dimensions and lung volume. Consequently, altered postoperative lung capacity leads to atelectasis, restricted ventilatory impairment, and diaphragmatic dysfunction [24]. Postoperative pulmonary complications are characterized by impaired pulmonary gas exchange, which appears within the first few days after surgery and may last up to 7 days. Postoperative pulmonary complications include hypoxemia, atelectasis, pleural effusion, as well as prolonged mechanical ventilation or extubation failure. Abdominal surgery requires transection of the abdominal muscles, and damage to the chest wall from surgical procedures leads to diaphragmatic dysfunction [25]. The impact of postoperative pulmonary complications on mortality is higher in patients undergoing thoracic surgery than in those undergoing abdominal surgery [26]. The risk index for postoperative respiratory complications (ARISCAT score) has been developed based on a population-based study that included patients undergoing surgery with general and regional anesthesia [27]. However, postoperative treatment and data at extubation were not included as predictors in the risk index. Extubation failure is consistently associated with increased morbidity and mortality in postoperative patients requiring mechanical ventilation [28, 29]. Reintubation outside the operating room is likely to result in increased procedural complications, such as hypoxia, hypotension, arrhythmias, and aspiration, compared to initial intubation [30, 31]. In addition, patients with extubation failure have been shown to have higher postoperative complications, including myocardial infarction and acute renal failure, as well as more blood transfusions due to bleeding complications [32, 33].

Due to the significance of postoperative patients in the current clinical practice of intensive care and the impact of reintubation on the prognosis in these patients, we aimed to investigate the clinical predictors of reintubation in postoperative patients.

## Methods

### Study participants

We conducted a post-hoc analysis of a multicenter prospective observational study across five tertiary care centers in Japan [34]. The original study included adult patients who underwent invasive mechanical ventilation for > 24 h between May 2017 and April 2019. The patients were extubated after successful SBT and cuff leak test. According to the weaning strategies with national consensus [35], SBT was performed for 30 min on positive end-expiratory pressure (PEEP) of 5 cmH_2_O with pressure support (PS) of 5 cmH_2_O. The risk of upper airway obstruction was examined by the cuff leak test before extubation and confirmed as low risk with a cuff leak volume > 110 mL and a percentage of cuff leak > 10%. The extubation protocol for the study patients is presented in detail the Additional file 1, Table S1. Patients younger than 18 years; those who were tracheotomized, discharged or died with mechanical ventilation; those extubated at the discretion of other than the prescribed procedure; those receiving extracorporeal membrane oxygenation, and those who died within 48 h after extubation were excluded. Based on continuous monitoring after extubation, reintubation and the use of noninvasive respiratory support were determined and performed by the intensivist in accordance with standard practice. The present study was approved by the ethics review board of Osaka University Hospital (Approval Number: 22247), and the requirement for written informed consent was waived.

### Details of data collected

The present study included postoperative patients from the original cohort and utilized data from those patients. We collected the patient characteristics of this cohort, including age, sex, body mass index, Acute Physiology and Chronic Health Evaluation (APACHE) II score, comorbidities, and systemic diagnosis for ICU admission. For clinical convenience and validity, respiratory and cardiovascular complications were presented as a single variable (underlying respiratory disease or pneumonia occurrence during mechanical ventilation, and underlying or new occurrence of heart failure during mechanical ventilation). Underlying and new occurrence of heart failure, defined as New York Heart Association functional classification IV or left ventricular ejection fraction ≤ 40%, was derived from a comorbid diagnosis and diagnosis at the time of initiation of mechanical ventilation. Comorbidity of chronic obstructive pulmonary disease (COPD), asthma, and other respiratory diseases (restrictive or obstructive lung diseases) integrated as underlying respiratory disease. The occurrence of pneumonia was identified as both the diagnosis at the time of initiation of mechanical ventilation and pneumonia occurrence based on observations made during mechanical ventilation. As processes of care during mechanical ventilation, the duration of mechanical ventilation was documented until extubation was attempted. Arterial blood gas and respiratory data during successful SBT were obtained at least 15 min after the commencement of the SBT. Parameters before extubation included Sequential Organ Failure Assessment score and the GCS score prior to extubation, as well as fluid balance and number of endotracheal suctioning episodes during the 24 h before extubation. As respiratory support after extubation, reintubation and the use and cause of noninvasive respiratory support (noninvasive ventilation [NIV] and high-flow nasal cannula [HFNC]) within 48 h after extubation were collected. The ICU and hospital length of stay, and the ICU and hospital mortality rates were recorded as patient outcomes.

### Outcomes measured

The primary outcome of this study was reintubation within 48 h after extubation. The secondary outcomes were lengths of ICU and hospital stay, and ICU and hospital mortality.

### Statistical analyses

Numerical data are presented as medians with interquartile ranges (IQRs) and categorical data as numbers and percentages. To delineate the distribution among event categories, the Mann–Whitney U test was applied to continuous variables and the chi-square test or Fisher’s exact test to categorical variables. The association between potential clinical predictors (systemic diagnosis, respiratory and cardiovascular complications, rapid shallow breathing index, fluid balance, the number of endotracheal suctioning episodes during the 24 h before extubation, and duration of mechanical ventilation) and reintubation were separately investigated by logistic regression analyses [11, 26, 34, 36]. To adjust for potential confounders, age, GCS score before extubation, and PaO_2_:FiO_2_ during SBT were considered in the regression models [9]. A visual description of the nonlinear relationships between the number of endotracheal suctioning episodes during the 24 h before extubation and the estimated probability of reintubation was presented using restricted cubic splines with 3 knots in the logistic regression model. Restricted cubic splines are a flexible statistical method for modelling non-linear relationships between a continuous predictive variable and the log odds of the outcome in regression models. They allow for smooth curve fitting while maintaining stability at the tails of the predictor distribution, reducing the risk of overfitting. Moreover, we performed a subgroup analysis according to the use of noninvasive respiratory support after extubation, considering the potential impact on the risk of reintubation. Subgroup analyses were also conducted in the two groups based on the median APACHE II score in this cohort or stratified by the risk of extubation failure. Patients older than 65 years and those with cardiopulmonary complications (COPD or chronic heart failure) were considered at a high risk for extubation failure [2, 37]. The interaction effect was evaluated for statistically significant subgroup differences using the multivariable logistic regression model. A significance level of < 0.05 in a two-tailed test was considered statistically significant. All analyses were performed using R, version 4.3.2 (R Foundation for Statistical Computing, Vienna, Austria).

## Results

Following standardized extubation of 355 postoperative patients, 38 (10.7%) required reintubation within 48 h (Table 1, Additional file 1: Figure S1). Reintubation occurred at a median of 11.2 h (IQR 2.6–23.9) after attempted extubation. The patients who were successfully extubated and those who required reintubation had comparable patient backgrounds. Patients who required reintubation had underlying respiratory disease or pneumonia occurrence more commonly, had longer duration of mechanical ventilation before extubation, and had more frequent endotracheal suctioning compared to patients who were successfully extubated (Table 2). Regarding respiratory support after extubation, reintubated patients more frequently recieved NIV alone, HFNC alone, or both NIV and HFNC compared to patients with successful extubation. The indications for NIV or HFNC were mostly refractory hypoxemia or prophylactic (Additional file 1: Table S1). As for patient outcomes, patients who required reintubation had similar mortality rates with significantly longer ICU and hospital stays compared to those who had successful extubation (17.12 [12.81–28.23] vs. 6.56 [3.95–10.70] d, and 70 [49–127] vs. 40 [27–73] d, *p* < 0.001 for both) (Table 3).

**Table 1:** Patient characteristics stratified by extubation outcomes

	Total cohort (*n* = 355)	Successful extubation (*n* = 317)	Reintubation (*n* = 38)	*P* value
Age, years	69 (55–76)	69 (54–76)	69.5 (63–76)	0.674
Male sex, *n* (%)	221 (62.3)	195 (61.5)	26 (68.4)	0.481
Body mass index, kg/m^2^	22.7 (20.1–25.6)	22.8 (20.1–25.9)	22.2 (19.7–24.1)	0.297
APACHE II score	17 (13–22)	17 (13–22)	16 (13–20)	0.407
Comorbidity, *n* (%)				
Heart failure	99 (27.9)	88 (27.8)	11 (28.9)	0.850
COPD	27 (7.6)	24 (7.6)	3 (7.9)	1.000
Asthma	16 (4.5)	14 (4.4)	2 (5.3)	0.684
Other respiratory diseases	39 (11.0)	31 (9.8)	8 (21.1)	0.051
Diabetes mellitus	96 (27.0)	83 (26.2)	13 (34.2)	0.334
Chronic kidney disease	74 (20.8)	64 (20.2)	10 (26.3)	0.399
Malignancy	36 (10.1)	29 (9.1)	7 (18.4)	0.087
Systemic diagnosis for ICU admission, *n* (%)				
Cardiac	224 (63.1)	199 (62.8)	25 (65.8)	0.413
Gastrointestinal	74 (20.8)	68 (21.5)	6 (15.8)	
Respiratory	24 (6.8)	18 (5.7)	6 (15.8)	
Neurological	12 (3.4)	12 (3.8)	0 (0.0)	
Renal urological	9 (2.5)	9 (2.8)	1 (2.6)	
Others	15 (4.2)	15 (4.7)	0 (0.0)	

Data are expressed as medians (interquartile range) or *n* (%)

*APACHE* Acute Physiology and Chronic Health Evaluation, *COPD* chronic obstructive pulmonary disease, *ICU* intensive care unit

**Table 2:** Processes of care during mechanical ventilation

	Total cohort	Successful extubation	Reintubation	*P* value
Underlying respiratory disease or pneumonia occurrence during mechanical ventilation, *n* (%)	108 (30.4)	89 (28.1)	19 (50.0)	0.008
Underlying or new occurrence of heart failure during mechanical ventilation, *n* (%)	105 (29.6)	94 (29.7)	11 (28.9)	1.000
Duration of mechanical ventilation, h	67.8 (42.5–126.5)	66.4 (42.1–118.2)	97.7 (63.9–164.6)	0.012
**ABG and respiratory data during successful SBT**				
pH	7.43 (7.40–7.46)	7.43 (7.40–7.46)	7.44 (7.41–7.46)	0.891
PaCO_2_, mmHg	40.70 (37.15–44.20)	40.60 (37.20–44.10)	42.95 (36.00–45.12)	0.252
PaO_2_, mmHg	106.0 (89.4–125.0)	105.5 (89.5–125.0)	106.5 (88.4–126.5)	0.797
Respiratory rate, breaths/min	18 (15–21)	18 (15–21)	18.5 (16–22)	0.150
Tidal volume, mL	417 (355–500)	422 (354–500)	408 (371–474)	0.549
PaO_2_:FiO_2_, mmHg	295 (232–353)	294 (234–353)	309 (213–351)	0.971
Rapid shallow breathing index, breaths/min/L	41.84 (32.50–56.05)	41.84 (31.82–56.18)	41.88 (35.54–55.54)	0.374
**Parameters before extubation**				
SOFA score	8 (6–10)	8 (6–10)	8 (6–10)	0.417
Fluid balance during the previous 24 h, mL	-327 (-1,088 to 421)	-327 (-1,064 to 400)	-412 (-1,198 to 404)	0.676
Glasgow Coma Scale score, point	11 (10–11)	11 (10–11)	11 (10–11)	0.189
Number of endotracheal suctioning episodes during the 24 h before extubation	12 (9–15)	12 (9–15)	15 (11–17)	0.009

Data are expressed as medians (interquartile range) or *n* (%)

*ABG* arterial blood gas, *SBT *spontaneous breathing trial, *SOFA* Sequential Organ Failure Assessment

**Table 3:** Respiratory support after extubation and patient outcomes

	Total cohort	Successful extubation	Reintubation	*P* value
**Use of noninvasive respiratory support during 48 h after extubation**				
NIV, *n* (%)	39 (11.0)	29 (9.1)	10 (26.3)	0.004
HFNC, *n* (%)	90 (25.4)	74 (23.3)	16 (42.1)	0.017
NIV or HFNC, *n* (%)	114 (32.1)	93 (29.3)	21 (55.3)	0.003
**Patient outcomes**				
ICU length of stay, d	7.39 (4.34–12.55)	6.56 (3.95–10.70)	17.12 (12.81–28.23)	< 0.001
Hospital length of stay, d	44 (28–79)	40 (27–73)	70 (49–127)	< 0.001
ICU mortality, *n* (%)	4 (1.1)	3 (0.9)	1 (2.6)	0.365
Hospital mortality, *n* (%)	22 (6.2)	18 (5.7)	4 (10.5)	0.275

Data are expressed as medians (interquartile range) or *n* (%)

*NIV* noninvasive ventilation, *HFNC* high-flow nasal cannula, *ICU* intensive care unit

### Potential clinical predictors of reintubation

**Fig. 1:**
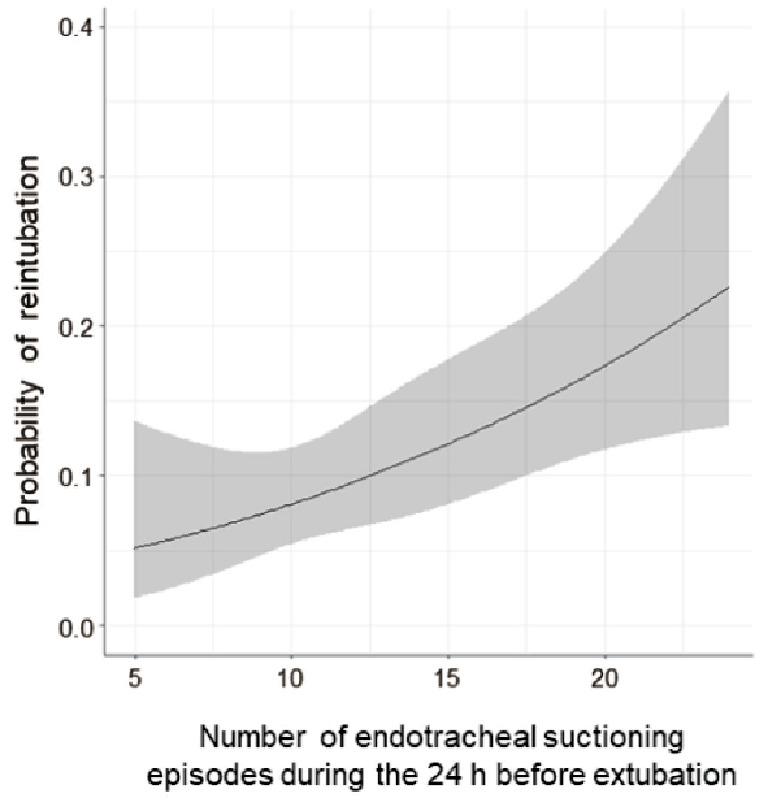
Association between reintubation and number of endotracheal suctioning episodes during the 24 h before extubation: restricted cubic spline curve of logistic regression analysis

The relationship between each potential clinical predictor and reintubation was investigated using logistic regression analysis (Table 4). Among the clinical predictors examined, underlying respiratory disease or pneumonia occurrence and the number of endotracheal suctioning episodes were found to be significantly associated with reintubation in the univariable analysis (crude odds ratio [OR] 2.56, 95% confidence interval [CI] 1.30–5.06, *p* = 0.007; crude OR 1.09, 95%CI 1.03–1.15, *p* = 0.002, respectively). The restricted cubic spline demonstrated that the unadjusted probability of reintubation significantly increased with increasing frequency of endotracheal suctioning (Fig. 1). After adjustment for fundamental confounding factors, the multivariable logistic regression model similarly showed that patients with underlying respiratory disease or pneumonia occurrence during mechanical ventilation had a higher risk of reintubation (adjusted OR 2.58, 95%CI 1.30–5.13, *p* = 0.007). The multivariable model consistently demonstrated the significant association between the number of endotracheal suctioning episodes during the 24 h before extubation and reintubation (adjusted OR 1.11, 95%CI 1.05–1.18, *p* < 0.001).

**Table 4:** Association between reintubation and clinical predictors: logistic regression analysis

	Crude OR (95%CI)	*P* value	Adjusted OR* (95%CI)	*P* value
Systemic diagnosis		0.691		0.581
Cardiac	4.02 (0.53–30.70)		4.98 (0.61–40.90)	
Gastrointestinal	2.82 (0.33–24.40)		3.65 (0.39–33.90)	
Respiratory	10.70 (1.19–95.70)		14.10 (1.46–135.00)	
Other	ref		ref	
Underlying respiratory disease or pneumonia occurrence during mechanical ventilation	2.56 (1.30–5.06)	0.007	2.58 (1.30–5.13)	0.007
Underlying or new occurrence of heart failure during mechanical ventilation	0.97 (0.46–2.03)	0.928	0.97 (0.46–2.04)	0.934
Rapid shallow breathing index	1.01 (0.99–1.02)	0.425	1.01 (0.99–1.02)	0.506
Fluid balance during the 24 h before extubation	1.00 (1.00–1.00)	0.843	1.00 (1.00–1.00)	0.780
Number of endotracheal suctioning episodes during the 24 h before extubation	1.09 (1.03–1.15)	0.002	1.11 (1.05–1.18)	< 0.001
Duration of mechanical ventilation	1.00 (0.99–1.00)	0.646	1.00 (0.99–1.00)	0.652

*OR* odds ratio, *CI* confidence interval

* Adjusted OR for age, Glasgow Coma Scale score, and PaO_2_:FiO_2_

### Subgroup analysis

During the 48 h after extubation, noninvasive respiratory support was used in 114 patients (32.1%) (Table 3). Consistently, patients with underlying respiratory disease or pneumonia occurrence had an increased risk of reintubation among the subgroup without noninvasive respiratory support (crude OR 4.00, 95%CI 1.45–11.00) and the subgroup with noninvasive respiratory support (crude OR 1.58, 95%CI 0.60–4.14) (Table 5). The number of endotracheal suctioning episodes during the 24 h before extubation also indicated a higher probability of reintubation among patients not requiring noninvasive respiratory support (crude OR 1.07, 95%CI 0.98–1.18) and patients requiring noninvasive respiratory support (crude OR 1.08, 95%CI 1.01–1.16). The associations between each independent variable and reintubation were not significantly different between the subgroups (p for interaction = 0.193 and 0.877). subgroup analysis according to the use of noninvasive respiratory support after extubation, considering the potential impact on the risk of reintubation. Moreover, the association between the identified clinical predictors and the rate of reintubation was consistent regardless of the severity of illness (p for interaction = 0.325 and 0.991) or risk of extubation failure (p for interaction = 0.777 and 0.084) (Additional file 1: Table S3, S4).

**Table 5:** Subgroup analysis of reintubation risk for each predictive variable, with and without noninvasive respiratory support after extubation

	Subgroup		*P* value for interaction
	Patients without noninvasive respiratory support (*n* = 241)	Patients with noninvasive respiratory support (*n* = 114)	
Reintubation, *n* (%)	17/241 (7.1)	21/114 (18.4)	
**Predictive variable**: Underlying respiratory disease or pneumonia occurrence			**0.193**
Crude OR (95%CI)	4.00 (1.45–11.00)	1.58 (0.60–4.14)	
*P* value	0.007	0.357	
**Predictive variable**: Number of endotracheal suctioning episodes during the 24 h before extubation			**0.877**
Crude OR (95%CI)	1.07 (0.98–1.18)	1.08 (1.01–1.16)	
*P* value	0.143	0.029	

*OR* odds ratio, *CI* confidence interval

## Discussion

### Key findings

In this multicenter prospective cohort of 355 postoperative critically ill patients, reintubation was performed at a rate of 10.7%. Our analysis investigated predictive factors for reintubation that are easily assessed in routine clinical practice for postoperative patients. A higher frequency of endotracheal suctioning and respiratory complications were significantly associated with increased reintubation rates. These factors were consistently associated with reintubation regardless of the use of noninvasive respiratory support after extubation.

### Relationship with prior studies

During mechanical ventilation, it is recommended that excessive airway secretions are removed by endotracheal suctioning as needed [38]. Pulmonary congestion resulting from excessive fluid infusion and airway inflammation lead to enhanced tracheobronchial secretions [39, 40]. Excessive airway secretions have been reported as a risk factor for upper airway obstruction and reintubation [41, 42], and the expert consensus in France and current weaning strategies with national consensus in Japan present decreased airway secretion clearance as an indicator of a high-risk group for extubation [35, 43]. However, the objective measure to assess the excessive amount of airway secretions has not been determined. In a prior single-center prospective observational study, we described that, as an objective measure of secretion, having > 15 endotracheal suctioning episodes/24 h was significantly associated with post-extubation stridor (adjusted OR 2.97, 95%CI 1.01–8.77) [36]. Furthermore, Haruna et al. have shown in a single-center retrospective study that endotracheal suctioning frequency (more than once every 2 h) is a contributing factor to reintubation in critically ill patients (OR 10.65, 95%CI 4.60–24.62) [44]. In this multicenter prospective study of postoperative patients using a standardized extubation procedure, we have clarified the validity of endotracheal suctioning frequency as an objective measure. As shown by the restricted cubic spline of the logistic regression analysis, the probability of reintubation increased significantly with the number of endotracheal suctioning episodes during the 24 h before extubation.

Underlying comorbidities have a significant impact on the postoperative outcomes of patients and the association between respiratory complications represented by COPD and reintubation has been reported [45, 46]. Patients with respiratory complications have chronic restrictions in the airflow and reduced pulmonary function. Thus, critically ill patients with respiratory complications often require prolonged mechanical ventilation, leading to respiratory muscle fatigue and impaired gas exchange, including suspended hypercapnia, which can result in an increased risk of post-extubation respiratory failure and extubation failure. In addition, pneumonia (including ventilator-associated pneumonia), which is one of the most frequently acquired infections in ICUs, is associated with prolonged mechanical ventilation and increased mortality. In a meta-analysis of 38 studies involving 22,304 critically ill patients, Li et al. demonstrated that patients with COPD and pneumonia had significantly higher rates of reintubation than those without COPD and pneumonia (OR 1.34, 95%CI 1.05–1.72; OR 2.58, 95%CI 1.72–3.87, respectively) [11]. Regarding reintubated patients after general anesthesia, a recent meta-analysis has described causal patient characteristics, including COPD [47]. Consistently, the present analysis indicates respiratory complications as an independent predictor of reintubation.

The use of noninvasive respiratory support after post-extubation respiratory failure is widely available, and international guidelines on liberation from mechanical ventilation contain recommendations for the use of prophylactic noninvasive respiratory support after extubation in high-risk patients with respiratory and cardiovascular complications [2, 48]. The use of noninvasive respiratory support has been shown to reduce the incidence of reintubation and postoperative respiratory failure compared to conventional oxygen therapy (COT), also in low-risk patients with the exception of older patients and those with severe illness [49]. Boscolo et al. reported a recent network meta-analysis of 5,063 patients from 32 trials evaluating noninvasive respiratory support (NIV, HFNC, or COT) after extubation in critically ill adult patients [50]. Compared with COT, NIV and HFNC were found to have lower reintubation rates (NIV: OR 0.61, 95%CI 0.46–0.81; HFNC: OR 0.60, 95%CI 0.43–0.84) along with lower incidences of ventilator-associated pneumonia, length of ICU and hospital stay, and hospital mortality. However, the advantages of noninvasive respiratory support vary depending on the clinical condition of the patient and the device type used. In this study, noninvasive respiratory support was used after extubation in 32.1% of the included patients. Patients who received noninvasive respiratory support in this study showed a higher rate of reintubation than those who did not. The risk of reintubation may have been exacerbated by the implementation of noninvasive respiratory support after the development of post-extubation respiratory failure with refractory hypoxemia. Nevertheless, subgroup analysis showed that the clinical predictors identified in our study were consistently associated with the rate of reintubation, regardless of the use of noninvasive respiratory support.

### Implications for clinicians

Endotracheal suctioning is an essential procedure in the management of mechanically ventilated patients, and endotracheal suctioning frequency can be assessed in clinical records without the use of specific measurement devices. Along with respiratory complications, endotracheal suctioning frequency is a clinically valid and versatile risk factor for reintubation. In postoperative patients who are commonly encountered in clinical practice, the predictors identified in this study may provide a definitive indicator of extubation.

### Strengths and limitations

The present study has several strengths. The findings constitute the analysis of data derived from a multicenter, prospective, observational study based on currently recommended extubation procedures. We applied a uniform SBT method with pressure support ventilation with low PS and PEEP levels, following international guidelines recommending inspiratory pressure augmentation [2]. However, our study has some limitations. A primary limitation is the observational nature of the study and the fact that the decision for post-extubation respiratory support, including reintubation, was made by the intensivist based on local standard practices. The reintubation rate in this study was comparable to those in existing reports of critically ill patients [4, 5], indicating that our cohort represents the standard clinical practice. Second, the implementation of endotracheal suctioning was not protocolized, and the need for endotracheal suctioning was determined by the clinician. The general guidelines are widely accepted for endotracheal suctioning procedures [38], and the standard procedures with periprocedural oxygenation as appropriate have been implemented. Third, the NIV or HFNC settings and devices used were not considered in this study. A recent systematic review indicated that the effect of noninvasive respiratory support differs depending on the presence of post-extubation respiratory failure and the risk of extubation failure [29]. Our data does not reveal the treatment strategies for noninvasive respiratory support, including the indications, device types, and settings. Fourth, the surgical procedure may affect postoperative outcomes including the need for reintubation, and our data did not include the detailed procedure. In the present study, multivariate logistic regression analysis showed no statistically significant differences in the probability of reintubation by disease among the included postoperative patients. However, the probability of reintubation has been reported to vary depending on the surgical procedure and the use of noninvasive respiratory support [51]. Thus, personalized extubation strategies are to be developed according to the surgical procedure and the pathophysiology of each patient. The predictive factors identified in this study were consistently and significantly associated with the rate of reintubation, as shown by detailed subgroup analyses, and may aid in the decision of extubation in the overall postoperative patients.

## Conclusions

In post-operative patients, endotracheal suctioning frequency and respiratory complications were independently associated with reintubation. Patients who underwent reintubation required prolonged treatment, and further large-scale investigations of this population are warranted.

## Electronic supplementary material

Below is the link to the electronic supplementary material.

Additional file 1: Table S1– Extubation protocol. Figure S1– Patient inclusion flowchart. Table S2– Indication of noninvasive respiratory support within 48 hours after extubation. Table S3– Association between reintubation and each predictive variable in the two groups based on median APACHE II score in this cohort. Table S4– Association between reintubation and each predictive variable stratified by risk of extubation failure

## Data Availability

The datasets of this analysis are available from the corresponding author upon reasonable request.

## Abbreviations

APACHE: Acute Physiology and Chronic Health Evaluation
CI: Confidence interval
COPD: Chronic obstructive pulmonary disease
COT: Conventional oxygen therapy
HFNC: High-flow nasal cannula
ICU: Intensive care unit
IQR: Interquartile ranges
GCS: Glasgow Coma Scale
NIV: Noninvasive ventilation
OR: Odds ratio
PEEP: Positive end-expiratory pressure
PS: Pressure support
SBT: Spontaneous breathing trial

## References

[1] Frutos-Vivar F, Esteban A, Apezteguia C, Gonzalez M, Arabi Y, Restrepo MI, et al. Outcome of reintubated patients after scheduled extubation. J Crit Care. 2011;26:502–9. 10.1016/j.jcrc.2010.12.015. 21376523 10.1016/j.jcrc.2010.12.015

[2] Ouellette DR, Patel S, Girard TD, Morris PE, Schmidt GA, Truwit JD, et al. Liberation from mechanical ventilation: an official American college of chest physicians/american thoracic society clinical practice guideline: inspiratory pressure augmentation during spontaneous breathing trials, protocols minimizing sedation, and non-invasive ventilation immediately after extubation. Chest. 2017;151:166–80. 10.1016/j.chest.2016.10.036. 27818331 10.1016/j.chest.2016.10.036

[3] Schonhofer B, Geiseler J, Dellweg D, Fuchs H, Moerer O, Weber-Carstens S et al. Prolonged weaning: S2k guideline published by the German Respiratory Society. Resp. 2020;1-102. 10.1159/000510085 10.1159/000510085 33302267

[4] Miltiades AN, Gershengorn HB, Hua M, Kramer AA, Li G, Wunsch H. Cumulative probability and time to reintubation in U.S. ICUs. Crit Care Med. 2017;45:835–42. 10.1097/CCM.0000000000002327. 28288027 10.1097/CCM.0000000000002327 PMC5896308

[5] Burns KEA, Sadeghirad B, Ghadimi M, Khan J, Phoophiboon V, Trivedi V, et al. Comparative effectiveness of alternative spontaneous breathing trial techniques: a systematic review and network meta-analysis of randomized trials. Crit Care. 2024;28:194. 10.1186/s13054-024-04958-4. 38849936 10.1186/s13054-024-04958-4 PMC11162018

[6] Gao F, Yang LH, He HR, Ma XC, Lu J, Zhai YJ, et al. The effect of reintubation on ventilator-associated pneumonia and mortality among mechanically ventilated patients with intubation: A systematic review and meta-analysis. Heart Lung. 2016;45:363–71. 10.1016/j.hrtlng.2016.04.006. 27377334 10.1016/j.hrtlng.2016.04.006

[7] Ippolito M, Sardo S, Tripodi VF, Latronico N, Bignami E, Giarratano A, et al. Association between spontaneous breathing trial methods and reintubation in adult critically ill patients: A systematic review and network meta-analysis of randomized controlled trials. Chest. 2024;166:1020–1034. 10.1016/j.chest.2024.06.3773 10.1016/j.chest.2024.06.3773 38964674

[8] Dadam MM, Pereira AB, Cardoso MR, Carnin TC, Westphal GA. Effect of reintubation within 48 hours on mortality in critically ill patients after planned extubation. Respir Care. 2024;69:829–38. 10.4187/respcare.11077. 38772683 10.4187/respcare.11077 PMC11285505

[9] Thille AW, Richard JC, Brochard L. The decision to extubate in the intensive care unit. Am J Respir Crit Care Med. 2013;187:1294–302. 10.1164/rccm.201208-1523CI. 23641924 10.1164/rccm.201208-1523CI

[10] Lombardi FS, Cotoia A, Petta R, Schultz M, Cinnella G, Horn J. Prediction of extubation failure in intensive care unit: systematic review of parameters investigated. Minerva Anestesiol. 2019;85:298–307. 10.23736/S0375-9393.18.12627-7. 29991220 10.23736/S0375-9393.18.12627-7

[11] Li W, Zhang Y, Wang Z, Jia D, Zhang C, Ma X, et al. The risk factors of reintubation in intensive care unit patients on mechanical ventilation: A systematic review and meta-analysis. Intensive Crit Care Nurs. 2023;74:103340. 10.1016/j.iccn.2022.103340. 36369190 10.1016/j.iccn.2022.103340

[12] Liu Y, Wei LQ, Li GQ, Lv FY, Wang H, Zhang YH, et al. A decision-tree model for predicting extubation outcome in elderly patients after a successful spontaneous breathing trial. Anesth Analg. 2010;111:1211–8. 10.1213/ANE.0b013e3181f4e82e. 20841406 10.1213/ANE.0b013e3181f4e82e

[13] Tsai TL, Huang MH, Lee CY, Lai WW. Data science for extubation prediction and value of information in surgical intensive care unit. J Clin Med. 2019;8:1709. 10.3390/jcm8101709 10.3390/jcm8101709 PMC6833107 31627316

[14] Baptistella AR, Mantelli LM, Matte L, Carvalho M, Fortunatti JA, Costa IZ, et al. Prediction of extubation outcome in mechanically ventilated patients: development and validation of the extubation predictive score (ExPreS). PLoS ONE. 2021;16:e0248868. 10.1371/journal.pone.0248868. 33735250 10.1371/journal.pone.0248868 PMC7971695

[15] Otaguro T, Tanaka H, Igarashi Y, Tagami T, Masuno T, Yokobori S, et al. Machine learning for prediction of successful extubation of mechanical ventilated patients in an intensive care unit: A retrospective observational study. J Nippon Med Sch. 2021;88:408–17. 10.1272/jnms.JNMS.2021_88-508. 33692291 10.1272/jnms.JNMS.2021_88-508

[16] Jia Y, Kaul C, Lawton T, Murray-Smith R, Habli I. Prediction of weaning from mechanical ventilation using convolutional neural networks. Artif Intell Med. 2021;117:102087. 10.1016/j.artmed.2021.102087. 34127233 10.1016/j.artmed.2021.102087

[17] Parada-Gereda HM, Tibaduiza AL, Rico-Mendoza A, Molano-Franco D, Nieto VH, Arias-Ortiz WA, et al. Effectiveness of diaphragmatic ultrasound as a predictor of successful weaning from mechanical ventilation: a systematic review and meta-analysis. Crit Care. 2023;27:174. 10.1186/s13054-023-04430-9. 37147688 10.1186/s13054-023-04430-9 PMC10161591

[18] Bansal V, Smischney NJ, Kashyap R, Li Z, Marquez A, Diedrich DA, et al. Reintubation summation calculation: A predictive score for extubation failure in critically ill patients. Front Med (Lausanne). 2021;8:789440. 10.3389/fmed.2021.789440. 35252224 10.3389/fmed.2021.789440 PMC8891541

[19] Ha TS, Oh DK, Lee HJ, Chang Y, Jeong IS, Sim YS, et al. Liberation from mechanical ventilation in critically ill patients: Korean society of critical care medicine clinical practice guidelines. Acute Crit Care. 2024;39:1–23. 10.4266/acc.2024.00052. 38476061 10.4266/acc.2024.00052 PMC11002621

[20] Piriyapatsom A, Williams EC, Waak K, Ladha KS, Eikermann M, Schmidt UH. Prospective observational study of predictors of re-intubation following extubation in the surgical ICU. Respir Care. 2016;61:306–15. 10.4187/respcare.04269. 26556899 10.4187/respcare.04269

[21] Japanese Society of Intensive Care Medicine. JIPAD annual report 2021. The Japanese Intensive care PAtient Database. [accessed 10 November 2024]. https://www.jipad.org/report/past-report/321-report

[22] Uzman S, Yilmaz Y, Toptas M, Akkoc I, Gul YG, Daskaya H, et al. A retrospective analysis of postoperative patients admitted to the intensive care unit. Hippokratia. 2016;20:38–43. 27895441 PMC5074395

[23] Raffa JD, Johnson AEW, O’Brien Z, Pollard TJ, Mark RG, Celi LA, et al. The global open source severity of illness score (GOSSIS). Crit Care Med. 2022;50:1040–50. 10.1097/CCM.0000000000005518. 35354159 10.1097/CCM.0000000000005518 PMC9233021

[24] Canet J, Gallart L. Postoperative respiratory failure: pathogenesis, prediction, and prevention. Curr Opin Crit Care. 2014;20:56–62. 10.1097/MCC.0000000000000045. 24240985 10.1097/MCC.0000000000000045

[25] Avolio AW, Gaspari R, Teofili L, Bianco G, Spinazzola G, Soave PM, et al. Postoperative respiratory failure in liver transplantation: risk factors and effect on prognosis. PLoS ONE. 2019;14:e0211678. 10.1371/journal.pone.0211678. 30742650 10.1371/journal.pone.0211678 PMC6370207

[26] Serpa Neto A, Hemmes SN, Barbas CS, Beiderlinden M, Fernandez-Bustamante A, Futier E, et al. Incidence of mortality and morbidity related to postoperative lung injury in patients who have undergone abdominal or thoracic surgery: a systematic review and meta-analysis. Lancet Respiratory Med. 2014;2:1007–15. 10.1016/S2213-2600(14)70228-0. 10.1016/S2213-2600(14)70228-0 25466352

[27] Canet J, Gallart L, Gomar C, Paluzie G, Valles J, Castillo J, et al. Prediction of postoperative pulmonary complications in a population-based surgical cohort. Anesthesiology. 2010;113:1338–50. 10.1097/ALN.0b013e3181fc6e0a. 21045639 10.1097/ALN.0b013e3181fc6e0a

[28] Chen S, Zhang Y, Che L, Shen L, Huang Y. Risk factors for unplanned reintubation caused by acute airway compromise after general anesthesia: a case-control study. BMC Anesthesiol. 2021;21:17. 10.1186/s12871-021-01238-4. 33435881 10.1186/s12871-021-01238-4 PMC7802267

[29] Pettenuzzo T, Boscolo A, Pistollato E, Pretto C, Giacon TA, Frasson S, et al. Effects of non-invasive respiratory support in post-operative patients: a systematic review and network meta-analysis. Crit Care. 2024;28:152. 10.1186/s13054-024-04924-0. 38720332 10.1186/s13054-024-04924-0 PMC11077852

[30] Menon N, Joffe AM, Deem S, Yanez ND, Grabinsky A, Dagal AH, et al. Occurrence and complications of tracheal reintubation in critically ill adults. Respir Care. 2012;57:1555–63. 10.4187/respcare.01617. 22324979 10.4187/respcare.01617

[31] Elmer J, Lee S, Rittenberger JC, Dargin J, Winger D, Emlet L. Reintubation in critically ill patients: procedural complications and implications for care. Crit Care. 2015;19:12. 10.1186/s13054-014-0730-7. 25592172 10.1186/s13054-014-0730-7 PMC4328699

[32] Hayashi LY, Gazzotti MR, Vidotto MC, Jardim JR. Incidence, indication and complications of postoperative reintubation after elective intracranial surgery. Sao Paulo Med J. 2013;131:158–65. 10.1590/1516-3180.2013.1313440. 23903264 10.1590/1516-3180.2013.1313440 PMC10852106

[33] Acheampong D, Guerrier S, Lavarias V, Pechman D, Mills C, Inabnet W, et al. Unplanned postoperative reintubation following general and vascular surgical procedures: outcomes and risk factors. Ann Med Surg (Lond). 2018;33:40–3. 10.1016/j.amsu.2018.08.013. 30167302 10.1016/j.amsu.2018.08.013 PMC6108072

[34] Tanaka A, Kabata D, Hirao O, Kosaka J, Furushima N, Maki Y, et al. Prediction model of extubation outcomes in critically ill patients: A multicenter prospective cohort study. J Clin Med. 2022;11:2520. 10.3390/jcm11092520. 35566646 10.3390/jcm11092520 PMC9102390

[35] Japanese Society of Intensive Care Medicine. Protocol on weaning from mechanical ventilation. https://www.jsicm.org/publication/kokyuki_ridatsu1503.html. 2015 [accessed 10 November 2024].

[36] Tanaka A, Uchiyama A, Horiguchi Y, Higeno R, Sakaguchi R, Koyama Y, et al. Predictors of post-extubation stridor in patients on mechanical ventilation: a prospective observational study. Sci Rep. 2021;11:19993. 10.1038/s41598-021-99501-8. 34620954 10.1038/s41598-021-99501-8 PMC8497593

[37] Jung B, Vaschetto R, Jaber S. Ten tips to optimize weaning and extubation success in the critically ill. Intensive Care Med. 2020;46:2461–3. 10.1007/s00134-020-06300-2. 33104823 10.1007/s00134-020-06300-2 PMC7585833

[38] Blakeman TC, Scott JB, Yoder MA, Capellari E, Strickland SL. AARC clinical practice guidelines: artificial airway suctioning. Respir Care. 2022;67:258–71. 10.4187/respcare.09548. 35078900 10.4187/respcare.09548

[39] Rogers DF. Physiology of airway mucus secretion and pathophysiology of hypersecretion. Respir Care. 2007;52:1134–49. 17716382

[40] Solymosi EA, Kaestle-Gembardt SM, Vadasz I, Wang L, Neye N, Chupin CJ, et al. Chloride transport-driven alveolar fluid secretion is a major contributor to cardiogenic lung edema. Proc Natl Acad Sci U S A. 2013;110:E2308–16. 10.1073/pnas.1216382110. 23645634 10.1073/pnas.1216382110 PMC3690871

[41] Jaber S, Quintard H, Cinotti R, Asehnoune K, Arnal JM, Guitton C, et al. Risk factors and outcomes for airway failure versus non-airway failure in the intensive care unit: a multicenter observational study of 1514 extubation procedures. Crit Care. 2018;22:236. 10.1186/s13054-018-2150-6. 30243304 10.1186/s13054-018-2150-6 PMC6151191

[42] Michetti CP, Griffen MM, Teicher EJ, Rodriguez JL, Seoudi H, Liu C, et al. FRIEND or FOE: A prospective evaluation of risk factors for reintubation in surgical and trauma patients. Am J Surg. 2018;216:1056–62. 10.1016/j.amjsurg.2018.07.004. 30017306 10.1016/j.amjsurg.2018.07.004

[43] Quintard H, l’Her E, Pottecher J, Adnet F, Constantin JM, De Jong A, et al. Experts’ guidelines of intubation and extubation of the ICU patient of French society of anaesthesia and intensive care medicine (SFAR) and French-speaking intensive care society (SRLF): in collaboration with the pediatric association of French-speaking anaesthetists and intensivists (ADARPEF), French-speaking group of intensive care and paediatric emergencies (GFRUP) and intensive care physiotherapy society (SKR). Ann Intensive Care. 2019;9:13. 10.1186/s13613-019-0483-1. 30671726 10.1186/s13613-019-0483-1 PMC6342741

[44] Haruna J, Tatsumi H, Kazuma S, Sasaki A, Masuda Y. Frequent tracheal suctioning is associated with extubation failure in patients with successful spontaneous breathing trial: a single-center retrospective cohort study. JA Clin Rep. 2022;8:5. 10.1186/s40981-022-00495-7. 35024978 10.1186/s40981-022-00495-7 PMC8758876

[45] Ramachandran SK, Nafiu OO, Ghaferi A, Tremper KK, Shanks A, Kheterpal S. Independent predictors and outcomes of unanticipated early postoperative tracheal intubation after nonemergent, noncardiac surgery. Anesthesiology. 2011;115:44–53. 10.1097/ALN.0b013e31821cf6de. 21552116 10.1097/ALN.0b013e31821cf6de

[46] Lin HT, Ting PC, Chang WY, Yang MW, Chang CJ, Chou AH. Predictive risk index and prognosis of postoperative reintubation after planned extubation during general anesthesia: a single-center retrospective case-controlled study in Taiwan from 2005 to 2009. Acta Anaesthesiol Taiwan. 2013;51:3–9. 10.1016/j.aat.2013.03.004. 23711598 10.1016/j.aat.2013.03.004

[47] Xie Z, Liu J, Yang Z, Tang L, Wang S, Du Y, et al. Risk factors for post-operative planned reintubation in patients after general anesthesia: A systematic review and meta-analysis. Front Med (Lausanne). 2022;9:839070. 10.3389/fmed.2022.839070. 35355600 10.3389/fmed.2022.839070 PMC8959864

[48] Rochwerg B, Einav S, Chaudhuri D, Mancebo J, Mauri T, Helviz Y, et al. The role for high flow nasal cannula as a respiratory support strategy in adults: a clinical practice guideline. Intensive Care Med. 2020;46:2226–37. 10.1007/s00134-020-06312-y. 33201321 10.1007/s00134-020-06312-y PMC7670292

[49] Hernandez G, Vaquero C, Gonzalez P, Subira C, Frutos-Vivar F, Rialp G, et al. Effect of postextubation high-flow nasal cannula vs conventional oxygen therapy on reintubation in low-risk patients: A randomized clinical trial. JAMA. 2016;315:1354–61. 10.1001/jama.2016.2711. 26975498 10.1001/jama.2016.2711

[50] Boscolo A, Pettenuzzo T, Sella N, Zatta M, Salvagno M, Tassone M, et al. Noninvasive respiratory support after extubation: a systematic review and network meta-analysis. Eur Respir Rev. 2023;32:220196. 10.1183/16000617.0196-2022 10.1183/16000617.0196-2022 PMC10074166 37019458

[51] Gaspari R, Spinazzola G, Ferrone G, Soave PM, Pintaudi G, Cutuli SL, et al. High-Flow nasal cannula versus standard oxygen therapy after extubation in liver transplantation: A matched controlled study. Respir Care. 2020;65:21–8. 10.4187/respcare.06866. 31270177 10.4187/respcare.06866

