# Supplementary material for: Clinical predictors of extubation failure in postoperative critically ill patients: a post-hoc analysis of a multicenter prospective observational study
Source: BMC Anesthesiol. 2025 Mar 15;25:127. doi: 10.1186/s12871-025-02996-1 (PMC11909811; doi:10.1186/s12871-025-02996-1)
Supplement: Supplementary file 1 — Additional file 1: Table S1– Extubation protocol. Figure S1– Patient inclusion flowchart. Table S2– Indication of noninvasive respiratory support within 48 hours after extubation. Table S3– Association between reintubation and each predictive variable in the two groups based on median APACHE II score in this cohort. Table S4– Association between reintubation and each predictive variable stratified by risk of extubation failure [file 12871_2025_2996_MOESM1_ESM.docx]

**Table S1.** Extubation protocol

| **Study protocol of extubation** [1] |
| --- |
| Patients were screened every morning by clinicians at medical meetings and determined eligible for spontaneous breathing trial (SBT) if the underlying respiratory failure or cause of mechanical ventilation was resolved. Sufficient oxygenation, inspiratory effort, adequate respiratory pattern, and hemodynamic stability were confirmed before SBT. The patients were extubated after successful SBT and a low risk of upper airway obstruction was confirmed by a negative cuff leak test (CLT) result prior to extubation. |
| **Prescribed SBT** |
| Patients were considered intolerant and SBT was interrupted by the clinician if any of the following were observed: significant deterioration of oxygenation compared to before SBT, respiratory rate ≥ 30 breaths/min, heart rate ≥ 140 beats/min, arrhythmia, myocardial ischemia symptom, sustained increased blood pressure, or appearance of respiratory distress as defined by paradoxical breathing, use of accessory muscles, sweating, or agitation. SBT success was determined when the patient tolerated 30-min SBT with a PEEP of 5 cmH_2_O and pressure support of 5 cmH_2_O. |
| **CLT methods** |
| Patients were suctioned intraorally and intratracheally, and assist-control mode was used as the ventilation mode for the CLT. The expiratory tidal volume was measured with the endotracheal tube cuff inflated to the occlusion volume. The endotracheal tube cuff was deflated and the mechanical exhaled tidal volume was observed over the next six consecutive respiratory cycles. The average of the three lowest expiratory tidal volumes was obtained, ensuring that false values were excluded. The tidal volume difference between inflation and deflation of the endotracheal tube cuff was calculated and defined as the cuff leak volume. The difference between the expiratory volume when the endotracheal tube cuff was inflated and deflated was calculated and defined as the cuff leak volume. The percent cuff leak was calculated by dividing the cuff leak volume by the tidal volume with the inflated endotracheal tube cuff and multiplying by 100. The risk of upper airway obstruction, a negative CLT result, was confirmed with a cuff leak volume >110 mL and a percentage of cuff leak >10%. |
| *SBT* spontaneous breathing trial, *CLT* cuff leak test, *PEEP* positive end-expiratory pressure. |

**Figure S1.** Patient inclusion flowchart

**
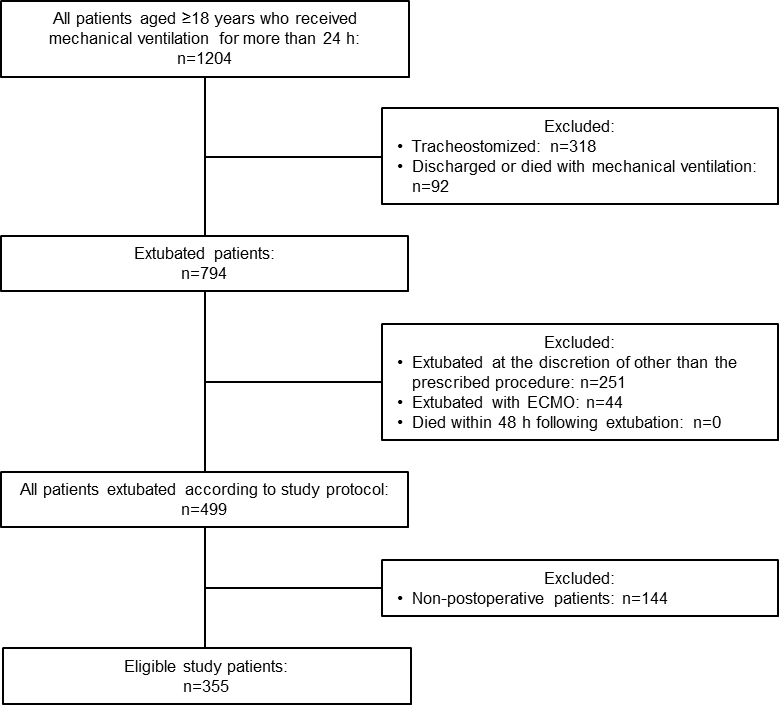
**

*ECMO* extracorporeal membrane oxygenation, *ICU* intensive care unit

**Table S2.** Indication of noninvasive respiratory support within 48 hours after extubation

|  | Total cohort  (n=355) | Successful extubation (n=317) | Reintubation  (n=38) |
| --- | --- | --- | --- |
| Cause of NIV use, n (%) |  |  |  |
| Refractory hypoxemia | 18/39 (46.2) | 12/29 (41.4) | 6/29 (60.0) |
| Increased breathing effort | 7/39 (17.9) | 4/29 (13.8) | 3/29 (30.0) |
| Heart failure | 1/39 (2.6) | 0/29 (0) | 1/29 (10.0) |
| Prophylactic | 13/39 (33.3) | 13/29 (44.8) | 0/29 (0) |
| Cause of HFNC use, n (%) |  |  |  |
| Refractory hypoxemia | 26/90 (28.9) | 18/74 (24.3) | 8/16 (50.0) |
| Increase of work of  breathing | 13/90 (14.4) | 8/74 (10.8) | 5/16 (31.2) |
| Heart failure | 3/90 (3.3) | 3/74 (4.1) | 0/16 (0.0) |
| Prophylactic | 48/90 (53.3) | 45/74 (60.8) | 3/16 (18.8) |
| Data are expressed as n (%).  *NIV* noninvasive ventilation, *HFNC* high-flow nasal cannula. | | | |

**Table S3.** Association between reintubation and each predictive variable in the two groups based on median APACHE II score in this cohort

|  | Subgroup | | P value for interaction |
| --- | --- | --- | --- |
|  | APACHE II score <17  (n=163) | APACHE II score ≥17  (n=192) |  |
| Reintubation, n (%) | 20/163 (12.3) | 18/192 (9.4) |  |
| **Predictive variable:** Underlying respiratory disease or pneumonia occurrence | | | **0.325** |
| Crude OR (95%CI) | 1.95 (0.72–5.29) | 4 (1.43–11.20) |  |
| P value | 0.193 | 0.008 |  |
| **Predictive variable:** Number of endotracheal suctioning episodes during the 24 h before extubation | | | **0.991** |
| Crude OR (95%CI) | 1.09 (1.01–1.17) | 1.09 (1.00–1.18) |  |
| P value | 0.022 | 0.052 |  |
| *APACHE* Acute Physiology and Chronic Health Evaluation, *OR* odds ratio, *CI* confidence interval. | | | |

**Table S4.** Association between reintubation and each predictive variable stratified by risk of extubation failure

|  | Subgroup | | P value for interaction |
| --- | --- | --- | --- |
|  | Patients without high risk of extubation failure (n=106) | High risk patients* (n=249) |  |
| Reintubation, n (%) | 10/106 (9.4) | 28/249 (11.2) |  |
| **Predictive variable:** Underlying respiratory disease or pneumonia occurrence | | | **0.777** |
| Crude OR (95%CI) | 3.00 (0.80–11.30) | 2.40 (1.08–5.32) |  |
| P value | 0.104 | 0.031 |  |
| **Predictive variable:** Number of endotracheal suctioning episodes during the 24 h before extubation | | | **0.084** |
| Crude OR (95%CI) | 1.16 (1.06–1.27) | 1.04 (0.96–1.13) |  |
| P value | 0.002 | 0.309 |  |
| *Patients older than 65 years and with cardiopulmonary complications (chronic obstructive pulmonary disease or chronic heart failure) were defined as being at higher risk for extubation failure.  *OR* odds ratio, *CI* confidence interval. | | | |

**References**

[1] Tanaka A, Kabata D, Hirao O, Kosaka J, Furushima N, Maki Y, et al. Prediction model of extubation outcomes in critically ill patients: A multicenter prospective cohort study. J Clin Med. 2022;11:2520. https://doi.org/10.3390/jcm11092520
